# Determination of MIC of amoxicillin among clinical strains of *Aggregatibacter actinomycetemcomitans* from various geographic regions endorse the continuous use of this drug for treatment of periodontitis

**DOI:** 10.1080/20002297.2017.1325251

**Published:** 2017-05-30

**Authors:** Anne Birkeholm Jensen, Dorte Haubek, Anders Johansson, Rolf Claesson, Niels Nørskov-Lauritsen

**Affiliations:** ^a^ Department of Dentistry, Aarhus University, Denmark; ^b^ Division of Molecular Periodontology, Department of Odontology, Faculty of Medicine, Umeå University, Umeå, Sweden; ^c^ Division of Oral Microbiology, Department of Odontology, Umeå University, Sweden; ^d^ Department of Clinical Microbiology, Aarhus University Hospital, Denmark

## Abstract

**Aim**: The combination of amoxicillin and metronidazole is used in the treatment of *Aggregatibacter actinomycetemcomitans (Aa)*-associated periodontitis in some patients. *Aa* is innately susceptible to betalactam agents, but resistance or decreased susceptibility has been reported. Elucidation of the prevalence and mechanisms of resistance to betalactams in *Aa* is important in order to treat periodontitis patients with the most optimal antibiotic regimen.

**Method**: 259 *Aa* strains were collected from patients with geographically diverse origin and serotyped with a multiplex PCR. Minimal inhibitory concentration (MIC) values were determined using the agar dilution method, with amoxicillin (range 0.00 mg/L to 8.00 mg/L) incorporated into 5 % blood agar plates with 20 mg/mL NAD. The plates were inoculated with 1 μL bacterial suspensions (10^4^ CFU/ml), incubated in 5 % CO_2_ at 37 C°, and visually inspected after 24 and 48 hours. A MIC ≥ 4.00 mg/L was categorised as resistant, using EUCAST interpretative criteria for *Haemophilus* species.

**Results**: None of 259 clinical *Aa* strains exhibited resistance towards amoxicillin. MIC-values varied from 0.25 mg/L to 2.00 mg/L. There was no clear correlation between MIC-value and serotype.

**Conclusion**: The study showed no contraindications to the continuous use of amoxicillin as a part of the treatment strategy in patients with *Aa*-associated periodontitis.

